# Declining Rates of Surgery for Nonmalignant Colorectal Polyps in the United States Over the Last Decade: A Real-World Analysis of Over 1 Million Patients

**DOI:** 10.1055/a-2926-6674

**Published:** 2026-09-01

**Authors:** Fouad Jaber, Ahmed-Jordan Salahat, Mohammad M. AlMasri, Kinan Obiedat, Mohammed Jaber, Wasseem Skef, Mai Khalaf, Tara Keihanian, Salmaan Jawaid, Mohamed Othman, Fares Ayoub

**Affiliations:** 1 Section of Gastroenterology, Hepatology & Nutrition 3989 Baylor College of MedicineHoustonTexasUnited States; 2 Department of Internal Medicine 24499 Manatee Memorial Hospital and Health SystemBradentonFloridaUnited States; 3 Department of Internal MedicineUniversity of Michigan Health-SparrowLansingUnited States; 4 Department of Medicine 5755 Yale UniversityNew HavenConnecticutUnited States; 5 Faculty of Medicine 123683 Al-Azhar UniversityGazaPalestine; 6 Section of Endoluminal Surgery and Interventional Gastroenterology 12339 University of Texas McGovern Medical SchoolHoustonTexasUnited States

**Keywords:** colon polyps, endoscopic resection, endoscopic mucosal resection, surgery

## Abstract

**Background** Despite advancements in endoscopic resection (ER), surgery for nonmalignant colorectal polyps remains common. A prior analysis reported increasing surgical rates from 2000 to 2014. Using the TriNetX US Research Network, we aimed to compare the proportion of patients referred for ER versus surgical colectomy for nonmalignant polyps each year from 2015 to 2023.

**Methods** We identified adults (≥18 years) diagnosed with nonmalignant colorectal polyps following colonoscopy using validated ICD-10 and CPT codes. Patients with colorectal cancer or inflammatory bowel disease were excluded. The primary outcome was incidence of ER (EMR or ESD) vs. colectomy between 2 weeks and 6 months post-colonoscopy. Secondary outcomes included 30-day adverse events (AEs) and mortality. Propensity score matching was used to compare groups. Odds ratios (ORs) were calculated for secondary outcomes.

**Results** Of 1,060,302 patients undergoing colonoscopy from 2015 to 2023, 6,295 (0.594%, 95% CI: 0.579–0.609%) were referred for ER and 4,208 (0.397%, 95% CI: 0.385–0.409%) were referred surgery for large nonmalignant colorectal polyps detected and not resected on index colonoscopy. ER incidence rose from 0.26% (2015) to 0.67% (2023), while surgery rates declined from 0.45% to 0.35%. Post-matching, ER was associated with significantly lower 30-day mortality (0.161% vs. 1.304%; OR: 0.122) and fewer AEs, including cardiac events, thromboembolic events, pulmonary complications, sepsis, and surgical site infections.

**Discussion** Over the past decade, ER for nonmalignant colorectal polyps increased while surgical resections declined. ER was associated with significantly lower mortality and morbidity. These findings support continued efforts to promote ER as a safer, effective alternative to surgery.

## Introduction

Nonmalignant colorectal polyps are commonly detected during routine screening colonoscopies and, while benign, require resection to mitigate the risk of malignant transformation. Historically, surgical resection was the primary approach for colorectal polyp removal. 1 However, over the past two decades, endoscopic resection (ER) techniques, including endoscopic mucosal resection (EMR) and endoscopic submucosal dissection (ESD), have become preferred due to their lower complication rates **,** shorter recovery times **,** and cost-effectiveness. 2 3 Studies confirm ER achieves comparable long-term efficacy to surgery in preventing colorectal cancer, with advanced tools and training programs improving accessibility over the past decade. 4 5

Despite these advancements, earlier analyses revealed a paradoxical rise in surgical resection rates for nonmalignant polyps from 2000 to 2014. 6 This trend raised concerns about overuse of invasive procedures **,** particularly given guidelines advocating endoscopic referral for complex polyps. 3 However, emerging evidence suggests a reversal: recent data from integrated healthcare systems show that colectomy rates for nonmalignant polyps declined by 90% between 2008 and 2018, coinciding with expanded endoscopic expertise and standardized referral pathways. 5 This divergence highlights the need to reassess national practice patterns in the context of modern endoscopic advancements.

Using the TriNetX US Research Network, a multi-institutional database aggregating electronic health records from 69 healthcare organizations, we aimed to determine the proportion of patients referred for ER versus surgical colectomy for nonmalignant polyps each year from 2015 to 2023 and to compare morbidity and mortality between both approaches.

## Methods

### Data Source

This retrospective cohort study utilized the TriNetX US Research Network, a large, multi-institutional database that aggregates de-identified electronic health records (EHRs) from 69 healthcare organizations across the United States, predominantly large academic medical centers and integrated health systems. Smaller community hospitals and outpatient endoscopy centers are underrepresented in the network. The database integrates both structured data, such as diagnoses and procedures, and unstructured clinical notes processed through natural language processing algorithms. Data quality is ensured through rigorous extraction protocols, and only aggregated, de-identified data are accessible to researchers, maintaining compliance with HIPAA regulations. As the data are fully de-identified, this study was exempt from institutional review board approval and did not require informed consent. Because TriNetX aggregates de-identified data at the network level from existing EHRs rather than through prospective patient recruitment, individual-level refusal or opt-out cannot be tracked or reported; all patients meeting inclusion criteria within participating institutions during the study period are captured in aggregate, without patient-level identifiers visible to investigators.

### Study Population and Design

In this study, a real-time search was conducted using the TriNetX platform extracting patient records spanning the years 2015 (debut year for CPT code for EMR), through 2023 (2024 excluded to avoid incomplete data bias at time of data pull).

We included patients (≥18 years) with nonmalignant polyps found on colonoscopy who underwent either EMR, ESD, or surgical removal identified using validated International Classification of Disease, Tenth Revision, Clinical Modification (ICD-10-CM) codes. Patients who underwent either EMR/ESD or surgical resection were identified using Current Procedural Terminology (CPT) codes. Due to lack of CPT code for ESD, we utilized HCPS code C977. Patients who had ICD-10-CM or CPT codes related to colorectal cancer or inflammatory bowel diseases were excluded. Records with missing procedural or outcome data were also excluded.

### Outcome Measures

#### Primary Outcome

This cohort included adult patients aged 18 years or older who underwent colonoscopy during the study period. Patients with any prior diagnosis of inflammatory bowel disease—such as Crohn’s disease or ulcerative colitis—were excluded. To qualify, patients were required to have a newly documented diagnosis of a nonmalignant colorectal polyp (including colon polyp, rectal polyp, or benign neoplasm of the colon or rectum) within one month following the index colonoscopy. Patients with any diagnosis of colorectal malignancy—including cancer of the colon, rectum, or rectosigmoid junction—were excluded. This cohort was designed to capture individuals with newly identified nonmalignant colorectal polyps that were not resected at the index colonoscopy.

The primary outcome was the proportion of patients in this cohort who underwent endoscopic treatment (either EMR or ESD) versus surgical colectomy, among those with a qualifying referral occurring between 14 days and 6 months following the index colonoscopy. To minimize misclassification of repeat procedures performed for the management of immediate postendoscopic complications or poor colonoscopy preparation, we excluded interventions occurring within the first two weeks following the index colonoscopy. This proportion was calculated separately for each calendar year from 2015 to 2023 to assess temporal trends in treatment approach. Further details regarding the cohort definitions, outcomes, index event, and time window definitions are described in **Supplementary File 1** and **Supplementary Table 1** .

#### Secondary Outcomes

For secondary outcomes, we performed a separate analysis selecting a comparison cohort that included all cases of ER versus surgery for large nonmalignant colorectal polyps. This cohort comprised adult patients (≥18 years) who underwent colonoscopy and were subsequently diagnosed with a nonmalignant colorectal polyp within one month of the procedure. Patients with a history of inflammatory bowel disease or any diagnosis of colorectal cancer were excluded.

Patients who underwent endoscopic treatment—either EMR or ESD—performed between 14 days and 6 months following the initial identification of the nonmalignant colorectal polyp on the index colonoscopy were part of the ‘endoscopic resection’ arm. Those patients who underwent colectomy during the same time frame following polyp identification were in the ‘colectomy’ cohort. Diagnostic and procedure codes and detailed definitions of the cohorts and secondary outcomes are provided in **Supplementary File 2, including Supplementary Tables 1 and 2** .

Secondary outcomes included 30-day adverse event (AEs) rates and mortality following ER versus surgery. Adverse events included cardiac complications, thromboembolic events, pulmonary complications, surgical site infections (SSIs), and gastrointestinal bleeding. Patients with cardiac complications were identified using ICD-10-CM code for “Acute myocardial infarction” (I21) or “Other cardiac arrhythmias” (I49). Pulmonary complications were identified using ICD-10-CM code for “Pulmonary embolism” (I26), “Other venous embolism and thrombosis” (I82), “acute respiratory distress syndrome” (J80), or “Respiratory failure, not elsewhere classified” (J96). SSIs were identified using the ICD-10-CM code for “Infection following a procedure” (T81.4). Bleeding complications were identified using ICD-10-CM code for “Other diseases of digestive system” (K92), “Hematemesis” (K92.0), “Melena” (K92.1), or “Gastrointestinal hemorrhage, unspecified” (K92.2). Finally, sepsis was identified using the ICD-10-CM code for “Other sepsis” (A41).

### Statistical Analysis

All statistical analyses were performed using the TriNetX Live platform (TriNetX LLC, Cambridge, MA), which provides browser-based, real-time analytic capabilities. Baseline characteristics of the study cohorts were summarized using proportions for categorical variables and means with standard deviations for continuous variables. To reduce the effect of measured confounding between the endoscopic and surgical resection groups, we performed one-to-one propensity score matching. Propensity score matching balances observed covariates between groups but does not address selection bias arising from cohort ascertainment, missing data, or loss to follow-up. The matching process included demographic variables such as age, gender, race, and ethnicity, as well as comorbidities including diabetes mellitus, hypertension, heart failure, chronic ischemic heart disease, chronic obstructive pulmonary disease, and chronic kidney disease. Use of anticoagulant and antiplatelet agents was also included in the matching algorithm.

The TriNetX platform utilizes logistic regression to generate propensity scores and applies a greedy nearest-neighbor algorithm with a caliper width of 0.1 pooled standard deviations, which has been shown to optimize balance between groups. The order of data rows is randomized to further reduce bias. Post-matching covariate balance was assessed using standardized mean differences (SMDs), with an SMD < 0.10 considered indicative of adequate balance; *p* -values were not used to assess balance, as they are influenced by sample size rather than the magnitude of imbalance. Following propensity score matching, odds ratios (ORs) for each secondary outcome were calculated within the matched cohort using logistic regression with treatment group (ER vs. surgery) as the sole predictor; these are referred to throughout as ORs rather than adjusted ORs, as covariate adjustment was achieved through matching rather than through additional regression adjustment. All point estimates, including yearly incidence proportions for the primary outcome, are reported with 95% confidence intervals calculated using the Wilson score method; given the large sample size. Statistical significance was defined as a two-sided *p* -value less than 0.05. Additional details on the propensity score methodology and analytical approach are provided in **Supplementary File 1** .

## Results

### Primary Outcomes: Temporal Trends in Treatment Approach

**Fig. 1:**
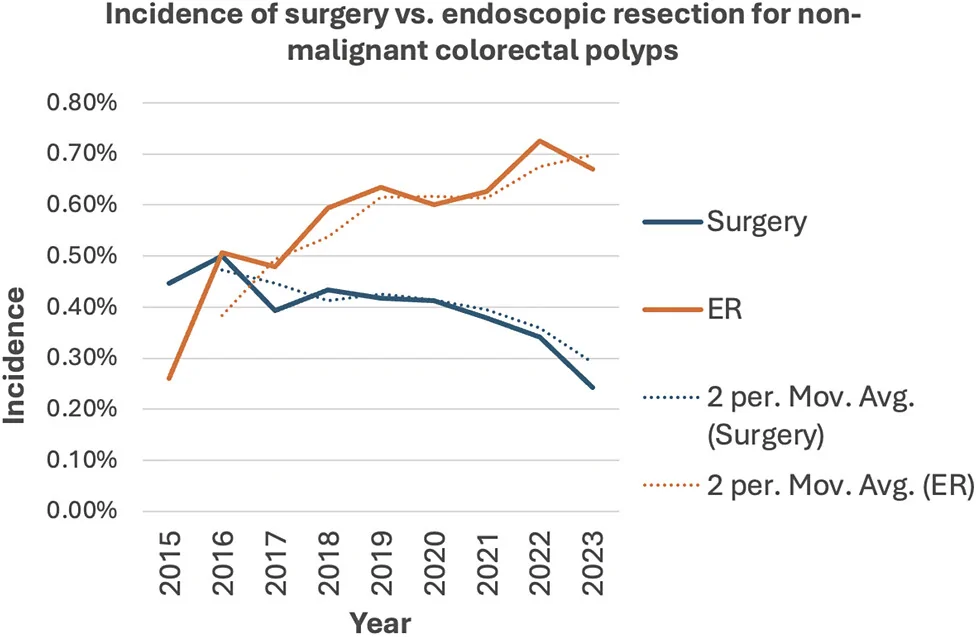
Trends for incidence of endoscopic resection versus surgery for nonmalignant colorectal polyps over the last decade.

For our primary outcome, a total of 1,060,302 patients underwent colonoscopy and found to have a nonmalignant colorectal polyp between 2015 and 2023 and among these, 6295 patients (0.594%, 95% CI: 0.579–0.609%) were referred for ER, and 4208 patients (0.397%, 95% CI: 0.385–0.409%) were referred surgical resection for nonmalignant colorectal polyps that were detected during the index colonoscopy. The incidence of ER increased steadily over the study period, rising from 0.26% (210/80,552 cases; 95% CI: 0.228–0.298%) in 2015 to 0.67% (1,175/175,225 cases; 95% CI: 0.633–0.710%) in 2023, representing a relative increase of more than 2.5-fold. In contrast, the incidence of surgical resection declined from 0.447% (360/80,552 cases; 95% CI: 0.403–0.495%) in 2015 to 0.349% (612/175,225 cases; 95% CI: 0.323–0.378%) in 2023. Annual incidence proportions and 95% confidence intervals for all years (2015–2023) are shown in Table 1 and plotted in Fig. 1 .

**Table 1:** Annual incidence of endoscopic resection (EMR) and surgical resection for nonmalignant colorectal polyps, 2015–2023, with 95% confidence intervals (Wilson score method).

Year	Total Patients	EMR *n* (%)	EMR 95% CI	Surgery *n* (%)	Surgery 95% CI
2015	80,552	210 (0.261%)	0.228–0.298%	360 (0.447%)	0.403–0.495%
2016	82,833	419 (0.506%)	0.460–0.556%	414 (0.500%)	0.454–0.550%
2017	97,393	467 (0.480%)	0.438–0.525%	383 (0.393%)	0.356–0.435%
2018	101,063	601 (0.595%)	0.549–0.644%	439 (0.434%)	0.396–0.477%
2019	122,050	774 (0.634%)	0.591–0.680%	510 (0.418%)	0.383–0.456%
2020	95,801	575 (0.600%)	0.553–0.651%	395 (0.412%)	0.374–0.455%
2021	143,066	897 (0.627%)	0.587–0.669%	542 (0.379%)	0.348–0.412%
2022	162,319	1177 (0.725%)	0.685–0.768%	553 (0.341%)	0.313–0.370%
2023	175,225	1175 (0.671%)	0.633–0.710%	612 (0.349%)	0.323–0.378%
**Total**	**1,060,302**	**6295 (0.594%)**	**0.579–0.609%**	**4,208 (0.397%)**	**0.385–0.409%**

### Secondary Outcomes

#### Patient Characteristics

For secondary outcomes, using the above cohort definitions, we found 15,084 patients underwent ER, while 8490 patients underwent surgery before matching. Patients in the ER cohort were older on average at the time of index colonoscopy compared to those in the surgery cohort (mean age 64.4 vs 61.6 years, *p* < 0.001). The ER group had a lower proportion of White patients (70.4% vs 73.5%, *p* < 0.001) and a higher proportion of Asian patients (2.4% vs 1.2%, *p* < 0.001). Hispanic patients were less likely to undergo ER compared to surgery (5% vs 7.8%, *p* < 0.001). The surgery cohort had a higher prevalence of comorbidities, including hypertension (59.6% vs 53.4%, *p* < 0.001), diabetes mellitus (24.6% vs 22.9%, *p* = 0.007), and COPD (12.3% vs 11%, *p* = 0.012). Differences in heart failure, chronic ischemic heart disease, chronic kidney disease, and use of anticoagulants or antiplatelet agents were not statistically significant. After propensity score matching, the two cohorts were well balanced across all measured variables, enabling valid comparative analysis ( Table 2 ).

**Table 2:** Baseline characteristics of cohorts before and after propensity score matching.

Baseline Variable	Before Matching			After Matching		
	ER Cohort	Surgery Cohort	SMD	ER Cohort	Surgery Cohort	SMD
**Number of patients**	15,084	8490	–	7,643	7,643	
**Mean Age at Index**	64.4 ± 10.6	61.6 ± 12.6	0.240	62.3 ± 10.9	62.1 ± 11.9	0.018
**Gender**						
Female	6297 (46.3%)	3078 (48.6%)	−0.046	2998 (48.3%)	3018 (48.6%)	−0.006
Male	6127 (45.0%)	2796 (44.2%)	0.016	2768 (44.6%)	2742 (44.1%)	0.010
**Race**						
Black or African American	1241 (9.1%)	588 (9.3%)	−0.007	554 (8.9%)	582 (9.4%)	−0.017
White	9586 (70.4%)	4652 (73.5%)	−0.069	4601 (74.1%)	4557 (73.4%)	0.016
Native Hawaiian or Other Pacific Islander	25 (0.2%)	11 (0.2%)	0.000	11 (0.2%)	10 (0.2%)	0.000
Asian	321 (2.4%)	79 (1.2%)	0.090	79 (1.3%)	79 (1.3%)	0.000
American Indian or Alaska Native	28 (0.2%)	14 (0.2%)	0.000	10 (0.2%)	14 (0.2%)	0.000
Unknown Race	2014 (14.8%)	857 (13.5%)	0.037	840 (13.5%)	845 (13.6%)	−0.003
Other Race	396 (2.9%)	126 (2.0%)	0.058	119 (1.9%)	125 (2.0%)	−0.007
**Ethnicity**						
Not Hispanic or Latino	9577 (70.4%)	4326 (68.4%)	0.043	4267 (68.7%)	4289 (69.0%)	−0.006
Hispanic or Latino	676 (5.0%)	493 (7.8%)	−0.115	444 (7.1%)	428 (6.9%)	0.008
Unknown Ethnicity	3358 (24.7%)	1508 (23.8%)	0.021	1501 (24.2%)	1495 (24.1%)	0.002
**Comorbidities**						
Diabetes mellitus	3114 (22.9%)	1558 (24.6%)	−0.040	1593 (25.6%)	1539 (24.8%)	0.018
Hypertensive diseases	7263 (53.4%)	3769 (59.6%)	−0.125	3802 (61.2%)	3717 (59.8%)	0.029
Heart failure	1159 (8.5%)	565 (8.9%)	−0.014	487 (7.8%)	558 (9.0%)	−0.043
Chronic ischemic heart disease	2373 (17.4%)	1139 (18.0%)	−0.016	1139 (18.3%)	1131 (18.2%)	0.003
Other chronic obstructive pulmonary disease	1504 (11.0%)	776 (12.3%)	−0.041	715 (11.5%)	774 (12.5%)	−0.031
Chronic kidney disease (CKD)	1488 (10.9%)	751 (11.9%)	−0.031	710 (11.4%)	744 (12.0%)	−0.019
Long-term anticoagulant/antiplatelet use	1194 (8.8%)	582 (9.2%)	−0.014	565 (9.1%)	577 (9.3%)	−0.007

Abbreviations: ER: endoscopic resection, SMD: standardized mean difference.

#### Secondary Outcomes: Mortality and 30-Day Adverse Events

The secondary outcomes of this study consisted of 30-day mortality and postprocedural AEs ( Table 3 ). In the matched cohorts, ER was associated with significantly lower 30-day mortality compared to surgery (0.161% vs 1.304%), corresponding to an 88% reduction in the odds of death (odds ratio [OR] 0.122, 95% CI: 0.063–0.236; *p* < 0.001). ER was also associated with lower rates of 30-day AEs. Cardiac complications occurred in 2.173% of ER patients versus 4.990% of surgical patients (OR 0.423; *p* < 0.001). Thromboembolic events were observed in 1.079% of the ER group compared to 3.123% in the surgery group (OR 0.338; *p* < 0.001). Pulmonary complications, including respiratory failure and pneumonia, were less frequent in the ER cohort (0.708% vs 4.250%; OR 0.161; *p* < 0.001). Sepsis occurred in 0.419% of ER patients versus 3.703% of surgical patients (OR 0.109; *p* < 0.001). Surgical site infections were reported exclusively in the surgery cohort (4.121%), with no cases in the ER group (risk difference: –4.121%; *p* < 0.001). The incidence of gastrointestinal bleeding was similar between groups (4.234% in ER vs 4.411% in surgery; OR 0.958; *p* = 0.47), indicating comparable risk for this adverse event.

**Table 3:** Mortality and 30-day adverse events between endoscopic resection and surgery.

Outcome	ER cohort (%, 95 CI)	Surgery cohort	Odds Ratio (OR)	95% Confidence Interval (CI)	*p* -Value
30-day Mortality	0.2 (0.090–0.274)	1.3 (1.077–1.589)	0.1	0.06 – 0.24	<0.001
Cardiac Events	2.2 (1.868–2.524)	5 (4.519–5.496)	0.4	0.34 – 0.52	<0.001
Thromboembolic Events	1.1 (0.865–1.330)	3.1 (2.760–3.541)	0.3	0.26 – 0.45	<0.001
Pulmonary Complications	0.7 (0.542–0.921)	4.3 (3.822–4.728)	0.2	0.12 – 0.23	<0.001
Sepsis	0.4 (0.297–0.590)	3.7 (3.302–4.150)	0.1	0.07 – 0.16	<0.001
Surgical Site Infection	N/A	4.1 (3.698–4.591)	–	–	<0.001
GI Bleeding	4.2 (3.810–4.714)	4.4 (3.971–4.893)	0.9	0.8 – 1.14	0.47

## Discussion

In this analysis of over 1 million US patients with nonmalignant colorectal polyps between 2015 and 2023, we found that rates of ER have gradually increased while rates of referral for surgical resection have decreased. We also find that surgical resection was associated with significantly higher morbidity and mortality compared to ER.

Earlier national analyses, such as the study by Peery et al., 6 observed increasing surgical resection rates for nonmalignant polyps between 2000 and 2014. This earlier pattern was attributed to various factors, such as limited access to trained endoscopists and inadequate institutional infrastructure causing increased referrals to high-volume centers for suspected malignant or technically difficult polyps. 6 Our study revealed that the incidence of ER increased from 0.26% in 2015 to 0.67% in 2023, a 2.5-fold rise, while surgical resections declined from 0.45% to 0.35%. These findings are consistent with recent national trends, which show a significant decrease in colectomy rates for benign neoplasms in the United States over the past decade. 7 For example, in a study using the national inpatient sample, the proportion of colectomies performed for benign compared to malignant disease declined from 28.6% in 2012 to 23.7% in 2019 ( *p* for trend < 0.001). 7 This pattern reflects a broader systemic shift toward less invasive management of benign colorectal polyps, with a marked increase in ER utilization and a modest but consistent reduction in surgical resections. The broader national adoption of ER observed in our study likely reflects improvements in physician training, institutional capabilities, and increased awareness of the benefits of minimally invasive, colon-preserving interventions. Societal initiatives promoting colorectal cancer screening may have also contributed to earlier detection and a greater proportion of polyps being managed endoscopically.

Endoscopic procedures were associated with a significantly reduced 30-day mortality rate than surgical resection in our study (0.161% vs 1.304%). This aligns with prior studies showing that ER is associated with lower mortality rates compared to surgical approaches (0.08% vs 0.7%). 8 In addition, our study revealed that ER also had a decreased incidence of postprocedural cardiac events (2.17% vs 4.99%), thromboembolic sequelae (1.08% vs 3.12%), pulmonary sequelae (0.71% vs 4.25%), and sepsis (0.42% vs 3.70%). These findings are consistent with previous research revealing that surgical approaches were associated with considerable risk for cardiovascular (2.1%), pulmonary (2.6%), and infectious events (3.6%). 9 The absence of surgical site infections in the ER group, compared to a 4.1% rate in the surgical group, further underscores the safety profile of endoscopic approaches. These findings are in line with meta-analyses and cohort studies demonstrating that ER is effective and cost-saving for large colorectal lesions, with fewer complications than surgery. 10

Bleeding remains the most common adverse event following ER, but our analysis found no significant difference in gastrointestinal bleeding rates between ER (4.23%) and surgery (4.41%). This may reflect advances in endoscopic technique and perioperative care, as well as the increasing ability to manage complications endoscopically without resorting to surgery. Notably, most bleeding and some perforation events after ER can now be treated endoscopically, further reducing the need for surgical intervention.

Despite these advantages, surgery remains necessary in select scenarios, such as nonlifting lesions, failed endoscopic attempts, or when deep submucosal invasion is suspected based on endoscopic optical diagnosis or histologic criteria. 11 For early colorectal cancer, some studies have reported higher curative resection rates with surgery, but without a significant difference in long-term disease-specific survival when compared to ER, especially for lesions limited to the mucosa or superficial submucosa. 12 Thus, individualized assessment remains essential, and multidisciplinary evaluation should guide management decisions.

A major strength of this study is the use of a large, nationally representative database, which enhances the generalizability of our findings and allows for the assessment of temporal trends across diverse health care settings. The application of propensity score matching minimized confounding and enabled valid comparisons of clinically relevant endpoints, including mortality and major complications.

However, several limitations should be acknowledged. The retrospective design and reliance on administrative coding introduce the potential for misclassification and underreporting of complications or procedures. However, the use of standardized coding (i.e., ICD-10 and CPT codes) facilitates consistent data capture among institutions. Also, institutional practice variation and patient referral variability may add variability, although the use of a national dataset mitigates this effect. The lack of granular procedural details, such as polyp size, morphology, and histopathology, limits the ability to assess technical factors influencing outcomes. Also, the exclusion of small hospitals from the TriNetX database may introduce selection bias, potentially limiting the applicability of our findings to larger, resource-rich centers. This exclusion is itself a source of selection bias that propensity score matching cannot correct, since matching balances measured covariates between the exposure groups within the cohort actually captured, but cannot address how that cohort was assembled in the first place. This also cautions against extrapolating our results to smaller US hospitals or to health care systems outside the United States, including those in Europe, without further validation. Similarly, because TriNetX does not report how many patients who underwent colonoscopy during the study period were excluded at each step (e.g., missing outcome data, incomplete follow-up), the extent of any further selection bias beyond the network’s institutional composition cannot be fully quantified. Finally, the TriNetX platform does not support graphical assessment of propensity score overlap (e.g., a Love plot) prior to matching, limiting formal verification of the common support assumption; we instead assessed post-matching covariate balance using standardized mean differences, all of which were below the 0.10 threshold conventionally used to indicate adequate balance ( Table 1 ), rather than relying on p-values, which are sensitive to sample size rather than the magnitude of imbalance.

In conclusions, our study demonstrates a clear and ongoing shift in the United States toward endoscopic management of nonmalignant colorectal polyps, with a corresponding decline in surgical resections. ER is associated with lower short-term mortality and complication rates compared to surgery, supporting its role as the preferred first-line treatment for most nonmalignant polyps. Continued efforts to expand access to advanced endoscopic techniques and to educate both physicians and patients are warranted to further reduce unnecessary surgeries and optimize outcomes for individuals with colorectal polyps.

**Abbreviations**
ER: Endoscopic Resection
EMR: Endoscopic Mucosal Resection
ESD: Endoscopic Submucosal Dissection
CPT: Current Procedural Terminology
ICD-10-CM: International Classification of Diseases, 10th Revision, Clinical Modification
HCPCS: Healthcare Common Procedure Coding System
EHR: Electronic Health Record
HIPAA: Health Insurance Portability and Accountability Act
AE: Adverse Event
SSIs: Surgical Site Infections
COPD: Chronic Obstructive Pulmonary Disease
OR: Odds Ratio
CI: Confidence Interval

## Data Availability

The data that support the findings of this study are not publicly available but are available from the corresponding author *initials (email)* upon reasonable request.

## Supplementary Material

Ergänzendes Material Supplementary Material

## References

[1] Zogg C K Rethinking priorities: cost of complications after elective colectomy Ann Surg 2016 264 02 312 322 26501705 10.1097/SLA.0000000000001511

[2] Kao K T Giap A Q Abbas M A Endoscopic excision of large colorectal polyps as a viable alternative to surgical resection Arch Surg 2011 146 06 690 696 21690445 10.1001/archsurg.2011.126

[3] Mann R Endoscopic Management of Complex Colorectal Polyps: current insights and future trends Front Med 2021 8 728704 10.3389/fmed.2021.728704 PMC8811151 35127735

[4] Aloysius M M National Trends in the incidence of sporadic malignant colorectal polyps in young patients (20-49 years): an 18-year SEER database analysisMedicina2024 60(04) 10.3390/medicina60040673 PMC11052004 38674319

[5] Alam A Declining colectomy rates for nonmalignant colorectal polyps in a large, ethnically diverse, community-based population Clin Transl Gastroenterol 2022 13 05 e00477 35347095 10.14309/ctg.0000000000000477 PMC9132519

[6] Peery A F Increasing rates of surgery for patients with nonmalignant colorectal polyps in the United States Gastroenterology 2018 154 05 1352 1360e3 29317277 10.1053/j.gastro.2018.01.003 PMC5880740

[7] Sakowitz S Decreasing rates of colectomy for benign neoplasms: a nationwide analysis PLoS One 2023 18 10 e0293389 37878628 10.1371/journal.pone.0293389 PMC10599571

[8] Peery A F Morbidity and mortality after surgery for nonmalignant colorectal polyps Gastrointest Endosc 2018 87 01 243 250e2 28408327 10.1016/j.gie.2017.03.1550 PMC5634910

[9] Ma C Morbidity and mortality after surgery for nonmalignant colorectal polyps: a 10-year Nationwide analysis Am J Gastroenterol 2019 114 11 1802 1810 31634261 10.14309/ajg.0000000000000407 PMC6830963

[10] Hassan C Efficacy and safety of endoscopic resection of large colorectal polyps: a systematic review and meta-analysis Gut 2016 65 05 806 820 25681402 10.1136/gutjnl-2014-308481

[11] Heitman S J Tate D J Bourke M J Optimizing resection of large colorectal polyps Curr Treat Options Gastroenterol 2017 15 01 213 229 28161819 10.1007/s11938-017-0131-5

[12] Silva G L Endoscopic versus surgical resection for early colorectal cancer-a systematic review and meta-analysis J Gastrointest Oncol 2016 7 03 326 335 27284463 10.21037/jgo.2015.10.02 PMC4880782

