# Supplementary material for: Declining Rates of Surgery for Nonmalignant Colorectal Polyps in the United States Over the Last Decade: A Real-World Analysis of Over 1 Million Patients
Source: Endosc Int Open. 2026 Sep 1;14:a29266674. doi: 10.1055/a-2926-6674 (PMC13551456; doi:10.1055/a-2926-6674)
Supplement: Supplementary file 1 [file 10-1055-a-2926-6674_29267608.pdf]

Supplemental Material

DECLINING RATES OF SURGERY FOR NON-MALIGNANT COLORECTAL POLYPS IN UNITED STATES OVER THE LAST DECADE: A REAL-WORLD ANALYSIS OF OVER 1 MILLION PATIENTS

Authors: Fouad Jaber, MD, MS[1], Ahmed-Jordan Salahat, MD[2], Mohammad Mahmoud AlMasri, MD[3], Kinan Obiedat, MD[4], Mohammed Jaber, MD(c)[5], Wasseem Skef, MD[6], Mai Khalaf, MD[1], Tara Keihanian, MD[1], Salmaan Jawaid, MD[1], Mohamed Othman, MD[1], Fares Ayoub, MD[1]

Table of Contents:

Supplementary File 1: Description of the cohorts for the primary outcome.

Supplementary File 2: Description of the cohorts for the secondary outcomes.

Supplementary Table 1. Codes used in the definition of cohorts.

Supplementary Table 2. Codes used in the definition of secondary outcomes.

Supplementary File 1: Description of the cohorts for the primary outcome.

This cohort consisted of adult patients aged 18 years or older who underwent a colonoscopy during the study period. Patients were excluded if they had any prior diagnosis of inflammatory bowel disease, including Crohn's disease or ulcerative colitis. To be included, patients were required to have a newly documented diagnosis of a non-malignant colorectal polyp—such as a colon polyp, rectal polyp, or benign neoplasm of the colon or rectum—within one month following the index colonoscopy. Patients with any diagnosis of colorectal malignancy, including cancer of the colon, rectum, or rectosigmoid junction, were excluded. This cohort was designed to capture individuals likely presenting with a newly identified, non-malignant colorectal polyp which was not resected at the index colonoscopy. For the primary outcome, we examined whether patients were referred for endoscopic treatment (EMR or ESD) versus referral for colectomy within 14 days to 6 months following the index colonoscopy and we trended the trend over time from 2015 till 2023. The cohorts were obtained from the TriNetX US Research Network, a large, multi-institutional database that aggregates de-identified electronic health records across the United States predominantly large academic medical centers and integrated health systems; smaller community hospitals and outpatient endoscopy centers are underrepresented in the network.

Index Event (Group 1)

Criterion	Description
**1A. Index Colonoscopy**	Adults (≥18 years) undergoing any of the following colonoscopy procedures:<br>- CPT: 45378, 45380, 45381, 45385, 45390, 44388, 44389, 44403, etc.<br>*(Full list below)*
**Exclusion**	Any prior diagnosis of:<br>- Crohn's Disease (ICD-10: K50)<br>- Ulcerative Colitis (ICD-10: K51)
**1B. Polyp Diagnosis**	Within **1 month** after index colonoscopy:<br>- ICD-10: K63.5 (colon polyp), K62.1 (rectal polyp), D12 (benign neoplasm of colon/rectum)
**Exclusion**	Any diagnosis of malignant colorectal neoplasm:<br>- ICD-10: C18, C19, or C20

Outcomes

Comparison of endoscopic resection (EMR/ESD) versus surgical resection (colectomy) over the period from 2015 to 2023, based on relevant procedural codes (listed below).

Supplementary File 2: Description of the cohorts for the secondary outcomes.

For secondary outcomes, we aimed to compare 30-day adverse events and mortality between the endoscopic resection cohort (cohort 1) and the surgical resection cohort (cohort 2)

Cohort 1: Endoscopic Resection (EMR/ESD)

This cohort included adult patients (≥18 years) who underwent colonoscopy and were subsequently diagnosed with a non-malignant colorectal polyp within one month of the procedure. Patients with a history of inflammatory bowel disease or any diagnosis of colorectal cancer were excluded. Among these, we identified the subgroup who were referred for endoscopic treatment—either endoscopic mucosal resection (EMR) or endoscopic submucosal dissection (ESD)—performed between 14 days and 6 months after the initial polyp diagnosis.

How did we code for Cohort 1?

Two groups were utilized to identify cohort 1 (Group 1 AND Group 2).

Group 1: Polyp Identification After Colonoscopy

Group 1 included adult patients aged 18 years or older who underwent a colonoscopy during the study period. Eligible procedures encompassed a broad range of diagnostic and therapeutic colonoscopies. Patients were excluded if they had any prior diagnosis of inflammatory bowel disease, specifically Crohn's disease or ulcerative colitis. To be included, patients needed to have a new diagnosis of a non-malignant colorectal polyp documented within one month following the colonoscopy. Patients with any diagnosis of colorectal cancer—such as malignancies of the colon, rectosigmoid junction, or rectum—were excluded. This group was intended to identify individuals likely found to have a non-malignant colorectal polyp following a colonoscopy.

Criterion	Description
**1A. Index Colonoscopy**	Adults (≥18 years) undergoing any of the following colonoscopy procedures:<br>- CPT: 45378, 45380, 45381, 45385, 45390, 44388, 44389, 44403, etc.<br>*(Full list below)*
**Exclusion**	Any prior diagnosis of:<br>- Crohn's Disease (ICD-10: K50)<br>- Ulcerative Colitis (ICD-10: K51)
**1B. Polyp Diagnosis**	Within **1 month** after index colonoscopy:<br>- ICD-10: K63.5 (colon polyp), K62.1 (rectal polyp), D12 (benign neoplasm of colon/rectum)
**Exclusion**	Any diagnosis of malignant colorectal neoplasm:<br>- ICD-10: C18, C19, or C20

Group 2: Endoscopic Treatment

Group 2 consisted of patients from Group 1 who subsequently underwent endoscopic resection for their polyp. This was defined as endoscopic mucosal resection (EMR) or endoscopic submucosal dissection (ESD) performed between 14 days and 6 months after the initial polyp diagnosis.

Criterion	Description
**2A. Polyp Diagnosis**	Same as Group 1B
**2B. Treatment (EMR/ESD)**	Within **14 days to 6 months** after 2A:<br>- CPT: 45390 (EMR), 45349, 44403<br>- HCPCS: C977 (Endoscopic Submucosal Dissection)

Cohort 2: Surgical Resection (Colectomy)

This cohort included adult patients (≥18 years) who underwent colonoscopy and were subsequently diagnosed with a non-malignant colorectal polyp within one month of the procedure. Patients with a history of inflammatory bowel disease or any diagnosis of colorectal cancer were excluded. Among these, we identified the subgroup who were referred for colectomy, performed between 14 days and 6 months after the initial polyp diagnosis.

<u>How did we code for Cohort 1?</u>

Two groups were utilized to identify cohort 1 (Group 1 AND Group 2).

Group 1: Polyp Identification After Colonoscopy

Same as Cohort 1, Group 1

Group 2: Surgical Treatment

Group 2 consisted of patients from Group 1 who subsequently underwent colectomy (partial or total) for their polyp. This was defined as surgical resection performed between 14 days and 6 months after the initial polyp diagnosis. Eligible procedures included partial and total colectomies, as identified by relevant procedural codes. This group aimed to capture patients who were managed surgically following the diagnosis of a non-malignant colorectal polyp.

Criterion	Description
**2A. Polyp Diagnosis**	Same as Group 1B
**2B. Treatment (Colectomy)**	Within **14 days to 6 months** after 2A:<br>- CPT: 44140, 44160, 44204, 44207, 44145, 44150, 44211, 45121, etc.<br>*(Full list below)*

Outcomes:
Secondary outcomes included 30-day adverse event (AEs) rates and mortality following ER versus surgery.

Adverse events included cardiac complications, thromboembolic events, pulmonary complications, surgical site infections (SSIs), and gastrointestinal bleeding. See Table 2 for the codes used.

Supplementary Table 1. Codes used in the definition of cohorts for secondary outcomes

**Group 1A: Colonoscopy Procedure Codes**	
**Code**	**Description**
1022231	Colonoscopy, flexible
45380	Colonoscopy, flexible; with biopsy, single or multiple
45378	Colonoscopy, flexible; diagnostic, including collection of specimen(s) by brushing or washing, when performed (separate procedure)
45381	Colonoscopy, flexible; with directed submucosal injection(s), any substance
1007534	Colonoscopy through stoma
44388	Colonoscopy through stoma; diagnostic, including collection of specimen(s) by brushing or washing, when performed (separate procedure)
45382	Colonoscopy, flexible; with control of bleeding, any method
44389	Colonoscopy through stoma; with biopsy, single or multiple
45386	Colonoscopy, flexible; with transendoscopic balloon dilation
45398	Colonoscopy, flexible; with band ligation(s) (eg, hemorrhoids)
45391	Colonoscopy, flexible; with endoscopic ultrasound examination limited to the rectum, sigmoid, descending, transverse, or ascending colon and cecum, and adjacent structures
45379	Colonoscopy, flexible; with removal of foreign body(s)
45393	Colonoscopy, flexible; with decompression (for pathologic distention) (eg, volvulus, megacolon), including placement of decompression tube, when performed
45389	Colonoscopy, flexible; with endoscopic stent placement (includes pre- and

	post-dilation and guide wire passage, when performed)
45392	Colonoscopy, flexible; with transendoscopic ultrasound guided intramural or transmural fine needle aspiration/biopsy(s), includes endoscopic ultrasound examination limited to the rectum, sigmoid, descending, transverse, or ascending colon and cecum, and adjacent structures
44391	Colonoscopy through stoma; with control of bleeding, any method
44404	Colonoscopy through stoma; with directed submucosal injection(s), any substance
44403	Colonoscopy through stoma; with endoscopic mucosal resection
44405	Colonoscopy through stoma; with transendoscopic balloon dilation
44390	Colonoscopy through stoma; with removal of foreign body(s)
44402	Colonoscopy through stoma; with endoscopic stent placement (including pre- and post-dilation and guide wire passage, when performed)
44408	Colonoscopy through stoma; with decompression (for pathologic distention) (eg, volvulus, megacolon), including placement of decompression tube, when performed
44407	Colonoscopy through stoma; with transendoscopic ultrasound guided intramural or transmural fine needle aspiration/biopsy(s), includes endoscopic ultrasound examination limited to the sigmoid, descending, transverse, or ascending colon and cecum and adjacent structures
44406	Colonoscopy through stoma; with endoscopic ultrasound examination, limited to the sigmoid, descending, transverse, or ascending colon and cecum and adjacent structures
45384	Colonoscopy, flexible; with removal of tumor(s), polyp(s), or other lesion(s) by hot biopsy forceps
45385	Colonoscopy, flexible; with removal of tumor(s), polyp(s), or other lesion(s) by snare technique
45381	Colonoscopy, flexible; with directed submucosal injection(s), any substance
45390	Colonoscopy, flexible; with endoscopic mucosal resection
45388	Colonoscopy, flexible; with ablation of tumor(s), polyp(s), or other lesion(s) (includes pre- and post-dilation and guide wire passage, when performed)
44403	Colonoscopy through stoma; with endoscopic mucosal resection
44401	Colonoscopy through stoma; with ablation of tumor(s), polyp(s), or other lesion(s) (includes pre-and post-dilation and guide wire passage, when performed)
**Group 1B/2A : Polyp Diagnosis Codes**	
**Code**	**Description**
K63.5	Polyp of colon
K62.1	Rectal polyp

D12	Benign neoplasm of colon, rectum, anus and anal canal
C20	Malignant neoplasm of rectum
**Excluded Diagnoses**	
**Code**	**Description**
K50	Crohn's disease [regional enteritis]
K51	Ulcerative colitis
C20	Malignant neoplasm of rectum
C18	Malignant neoplasm of colon
C19	Malignant neoplasm of rectosigmoid junction
**Group 2B (Cohort 1): EMR/ESD Procedure Codes**	
**Code**	**Description**
45390	Colonoscopy, flexible; with endoscopic mucosal resection
45349	Sigmoidoscopy, flexible; with endoscopic mucosal resection
44403	Colonoscopy through stoma; with endoscopic mucosal resection
HCPS code C977	Endoscopic Submucosal dissection
**Group 2B (Cohort 2):** Colectomy Codes	
**Code**	**Description**
1007455	Colectomy, partial
44140	Colectomy, partial; with anastomosis
44160	Colectomy, partial, with removal of terminal ileum with ileocolostomy
44143	Colectomy, partial; with end colostomy and closure of distal segment (Hartmann type procedure)
44145	Colectomy, partial; with coloproctostomy (low pelvic anastomosis)
1007463	Colectomy, total, abdominal, without proctectomy
44150	Colectomy, total, abdominal, without proctectomy; with ileostomy or ileoproctostomy
44141	Colectomy, partial; with skin level cecostomy or colostomy
44144	Colectomy, partial; with resection, with colostomy or ileostomy and creation of mucofistula
44146	Colectomy, partial; with coloproctostomy (low pelvic anastomosis), with colostomy
1007468	Colectomy, total, abdominal, with proctectomy

44155	Colectomy, total, abdominal, with proctectomy; with ileostomy
44158	Colectomy, total, abdominal, with proctectomy; with ileoanal anastomosis, creation of ileal reservoir (S or J), includes loop ileostomy, and rectal mucosectomy, when performed
44157	Colectomy, total, abdominal, with proctectomy; with ileoanal anastomosis, includes loop ileostomy, and rectal mucosectomy, when performed
44147	Colectomy, partial; abdominal and transanal approach
44151	Colectomy, total, abdominal, without proctectomy; with continent ileostomy
44156	Colectomy, total, abdominal, with proctectomy; with continent ileostomy
44207	Laparoscopy, surgical; colectomy, partial, with anastomosis, with coloproctostomy (low pelvic anastomosis)
44204	Laparoscopy, surgical; colectomy, partial, with anastomosis
44205	Laparoscopy, surgical; colectomy, partial, with removal of terminal ileum with ileocolostomy
44213	Laparoscopy, surgical, mobilization (take-down) of splenic flexure performed in conjunction with partial colectomy (List separately in addition to primary procedure)
44139	Mobilization (take-down) of splenic flexure performed in conjunction with partial colectomy (List separately in addition to primary procedure)
44210	Laparoscopy, surgical; colectomy, total, abdominal, without proctectomy, with ileostomy or ileoproctostomy
44208	Laparoscopy, surgical; colectomy, partial, with anastomosis, with coloproctostomy (low pelvic anastomosis) with colostomy
44206	Laparoscopy, surgical; colectomy, partial, with end colostomy and closure of distal segment (Hartmann type procedure)
44211	Laparoscopy, surgical; colectomy, total, abdominal, with proctectomy, with ileoanal anastomosis, creation of ileal reservoir (S or J), with loop ileostomy, includes rectal mucosectomy, when performed
44212	Laparoscopy, surgical; colectomy, total, abdominal, with proctectomy, with ileostomy

Supplementary Table 2. Codes used in the definition of secondary outcomes.

Outcome	Code	Description
**Pulmonary Embolism**	I26	Pulmonary embolism
	I82	Other venous embolism and thrombosis
**Pulmonary Events**	J80	Acute respiratory distress syndrome
	J96	Respiratory failure, not elsewhere classified
**Cardiac Events**	I21	Acute myocardial infarction
	I49	Other cardiac arrhythmias
**Surgical Site Infection**	T81.4	Infection following a procedure
**Sepsis**	A41	Other sepsis
	A41.9	Sepsis, unspecified organism
	A41.89	Other specified sepsis
**GI Bleeding**	K92.0	Hematemesis
	K92.1	Melena
	K92.2	Gastrointestinal hemorrhage, unspecified
	R58	Hemorrhage, not elsewhere classified
